# Impaired cerebrovascular reactivity is associated with disability and cognitive performance in relapsing–remitting multiple sclerosis

**DOI:** 10.1007/s10072-026-09163-5

**Published:** 2026-06-09

**Authors:** Evangelos Ntais, Vasiliki Kostadima, Andriana Haski, Konstantinos Tsamis, Sotirios Giannopoulos, Spyridon Konitsiotis

**Affiliations:** 1 Department of Neurology, Faculty of Medicine, School of Health Sciences, University of Ioannina, Ioannina, Greece; 2 Department of Neurology, University General Hospital of Ioannina, Ioannina, Greece; 3 Department of Physiology, Faculty of Medicine, School of Health Sciences, University of Ioannina, Ioannina, Greece; 4 Second Department of Neurology, School of Medicine, “Attikon” University Hospital, National and Kapodistrian University of Athens, Athens, Greece

**Keywords:** Cerebrovascular reactivity, Relapsing–remitting multiple sclerosis, Disability, Cognitive impairment, Breath-Holding Index

## Abstract

**Background and objectives:**

Cerebrovascular reactivity (CVR) reflects the ability of cerebral vessels to adapt to metabolic demands and may be impaired in multiple sclerosis (MS). Its clinical relevance in relapsing–remitting MS (RRMS), particularly in relation to disability and cognition, remains uncertain. We compared CVR, quantified by the Breath-Holding Index (BHI), between patients with RRMS and healthy controls, and examined its associations with neurological disability and cognitive performance.

**Methods:**

In this cross-sectional observational study consecutive RRMS patients and age- and sex-matched healthy controls (2:1 ratio) were enrolled. CVR to hypercapnia was assessed by transcranial Doppler using BHI. Cognitive performance was evaluated with the Brief International Cognitive Assessment for MS battery, and neurological disability with the Expanded Disability Status Scale (EDSS). Multivariable linear regression was used for adjusted analyses.

**Results:**

Ninety patients with RRMS and 45 healthy controls were included. BHI was lower in RRMS than in controls (0.88 ± 0.13 vs. 1.13 ± 0.13), with a mean difference of − 0.256 (95% CI − 0.302 to − 0.209). Lower BHI was associated with greater disability (EDSS: ρ=−0.420; 95% CI − 0.581 to − 0.228) and worse processing speed on the Symbol Digit Modalities Test (*r* = 0.50; 95% CI 0.33 to 0.64). In adjusted models, lower BHI remained independently associated with higher EDSS (B = − 3.51; 95% CI − 5.82 to − 1.20) and lower SDMT performance (B = 3.69; 95% CI 2.11 to 5.26).

**Discussion:**

RRMS patients exhibit reduced CVR, independently associated with disability and processing speed. Impaired CVR may represent a clinically relevant marker of disability and cognitive dysfunction in RRMS.

**Supplementary Information:**

The online version contains supplementary material available at 10.1007/s10072-026-09163-5.

## Introduction

Multiple sclerosis (MS) is a chronic autoimmune central nervous system disorder characterized by inflammation, demyelination and neurodegeneration. MS typically affects young adults and is characterized by physical disability, cognitive impairment, and other symptoms that affect quality of life [1]. Although the predominant view of MS is that of an autoimmune inflammatory condition, accumulating evidence indicates that vascular dysfunction and microvascular pathology may constitute integral components of MS pathobiology [2].

Current data suggest that the MS population has higher incidence of cardiovascular diseases when compared to age-matched healthy controls [3] and these factors may also contribute to disease progression and disability burden in MS. Epidemiological data also show that patients with MS have a higher risk of ischemic stroke [4]. Moreover, post-mortem studies have shown that MS patients exhibit a disproportionately increased burden of cerebral small vessel disease despite lower systemic atherosclerotic burden, and that periarteriolar inflammatory and degenerative changes represent key pathological features beyond classical demyelinating lesions, suggesting that cerebral microvascular pathology contributes to accelerated and irreversible disability progression [5].

Advanced perfusion MRI studies have consistently demonstrated diffuse cerebral hypoperfusion in MS, affecting both normal-appearing white matter and gray matter, with more pronounced abnormalities in progressive phenotypes and even at the earlier stages of the disease [6]. Importantly, perfusion abnormalities have been associated with greater physical disability and worse cognitive performance, and may precede structural atrophy, suggesting that chronic cerebral hypoperfusion represents a mechanistically relevant contributor to neurodegeneration and cognitive decline in MS [6, 7].

Cerebrovascular reactivity (CVR) reflects the ability of cerebral vasculature to regulate cerebral blood flow (CBF) and perfusion through changes in arterial resistance in response to vasoactive stimuli and constitutes a crucial component of neurovascular coupling [8]. In MS, inflammatory processes may induce endothelial dysfunction, impairing vasomotor regulation and predisposing to CVR failure, as the endothelium is involved in the regulation of vasomotor tone through the release of vasodilating agents [9]. Disruption of this autoregulatory mechanism may result in suboptimal perfusion during neuronal activation, promoting neuronal dysfunction and progressive tissue injury, which is evident in other neurodegenerative diseases [10]. Moreover, impaired CVR may disturb the integrity and efficient communication of functional networks, which are critical for the maintenance of normal cognitive performance [8].

CVR can be assessed using several techniques, including transcranial Doppler (TCD) sonography, dynamic susceptibility contrast and arterial spin labeling perfusion MRI during hypercapnic challenges, as well as blood oxygen level–dependent MRI–based hypercapnia paradigms, which enable the quantification of regional and global cerebrovascular responsiveness to vasoactive stimuli [11]. TCD sonography provides a noninvasive, convenient and real-time assessment of CVR using the Breath-Holding Index (BHI), which quantifies changes in CBF velocity in response to CO₂-mediated vasodilatory stimuli induced by breath holding [12].

Despite accumulating MRI evidence of hypoperfusion and emerging data on CVR abnormalities in MS, the extent to which CVR is impaired in relapsing–remitting MS (RRMS), its clinical relevance to disability accumulation and cognition, and its assessment using widely accessible TCD-based breath-holding paradigms remain insufficiently characterized. To date, no TCD-based study has demonstrated independent associations between CVR and both neurological disability and cognitive performance in a well-characterized RRMS cohort free of vascular comorbidities. Therefore, we aimed to (i) compare CVR between RRMS patients and age- and sex-matched healthy controls (HC) recruited at a 2:1 patient-to-control ratio using TCD-derived BHI and (ii) examine associations between CVR, neurological disability and cognitive performance.

## Materials and methods

### Study participants

This was a cross-sectional observational study. Participants were recruited at a single tertiary-care neurology center and were consecutive patients with the diagnosis of RRMS according to 2017 McDonald diagnostic criteria [1]. Sex- and age-matched healthy volunteers were recruited as a control group at a 2:1 ratio. Demographics, medical history, disease duration, current and previous disease-modifying therapies (DMTs), relapse activity (clinical or MRI-confirmed) within the preceding 2 years and neurological disability according to the Expanded Disability Status Scale (EDSS) [13] were recorded by an MS-specialist neurologist (VK).

Inclusion criteria were: (1) age between 18 and 55 years old, (2) diagnosis as RRMS based on the 2017 McDonald criteria, (3) no cardiovascular and metabolic diseases, (4) no psychiatric disorders and/or neurologic disease other than MS.

Exclusion criteria were: (1) clinical relapse or intravenous corticosteroid therapy within 3 months prior to evaluation, (2) pregnancy or breastfeeding, (3) extra- or intra-cranial arteries stenosis, (4) history of stroke, brain surgery, tumor, hypertension, diabetes mellitus, heart failure, (5) use of beta-blockers, calcium channel blockers or vasodilating agents (6) absence of TCD temporal acoustic windows.

To minimize selection bias, consecutive eligible patients were recruited. Neurosonologic and cognitive assessments were performed after demographic and clinical data collection and were blinded to these data and to each other.

### Cognitive assessment

Patients with RRMS underwent neuropsychological evaluation performed by a trained neurologist (A.H.). Cognitive function was assessed using the Brief International Cognitive Assessment for Multiple Sclerosis (BICAMS), which includes the Symbol Digit Modalities Test (SDMT), the learning trials of the Brief Visuospatial Memory Test–Revised (BVMT-R), and the Greek Verbal Learning Test (GVLT) [14]. Raw test scores were transformed into z-scores using age-, sex- and education-adjusted normative data in all tests [14–16]. Higher scores indicate better cognitive performance.

### CVR assessment

TCD examinations were performed in a quiet, air-conditioned room (23–26 °C) by a trained sonographer (EV). Cerebral hemodynamics was assessed using a TCD ultrasound system (Looki model, Atys Medical, Soucieu-en-Jarrest, France) through CVR to hypercapnia as measured by the BHI. Participants were instructed to refrain from caffeine and smoking for at least 12 h prior to the examination. Before testing, all participants were familiarized with breath-holding protocol, which consisted of an initial 5-min resting time followed by two 30-sec breath-holding maneuvers. Participants were instructed to avoid hyperventilation before breath holding and to perform a moderate inspiratory breath-holding to prevent a Valsalva episode.

Mean flow velocity (MFV) in the M1 segment (45–60 mm depth) of each middle cerebral artery was continuously recorded using bilateral 2-MHz probes fixed over the temporal windows with a stable insonation angle. Baseline MFV (BMFV) and post–breath-holding MFV (BH-MFV) were obtained before and immediately after each breath-holding maneuver, respectively.

BHI was calculated according to the following formula [12]:

$$\:\mathrm{BHI}=\frac{\left(\mathrm{BH-MFV}-\mathrm{BMFV}\right)}{\mathrm{BMFV}}\times\:\frac{100}{T}$$

BMFV(cm/s), BH-MFV (cm/s), T = duration of apnea(s).

Higher BHI values indicate greater CVR. For each participant, two measurements were obtained bilaterally, and the mean value was used for statistical analysis.

### Statistics

Statistical analyses were performed using IBM SPSS Statistics (version 27). The primary outcome was the difference in BHI between RRMS patients and HC. Secondary outcomes included associations between BHI, neurological disability, and cognitive performance. Data distributions were assessed visually and with the Shapiro–Wilk test. Continuous variables are presented as mean ± SD or median (IQR), as appropriate, and categorical variables as counts and percentages. Between-group comparisons between RRMS patients and HC were performed using independent-samples t tests (Welch correction when variances were unequal), and χ² tests for categorical variables. Within the RRMS cohort, associations between CVR (BHI) and clinical or demographic variables were assessed using Pearson or Spearman correlation analyses, as appropriate. To further characterize the healthy control group, the association between BHI and age was also examined using Pearson correlation analysis. Multivariable analyses were performed using hierarchical linear regression models, with EDSS modeled as a continuous outcome and model assumptions evaluated by residual diagnostics. Assumptions of linear regression, including linearity, normality of residuals, homoscedasticity, and influential observations, were evaluated. Unstandardized regression coefficients (B), effect sizes and 95% confidence intervals are reported. All tests were two-tailed, with statistical significance set at *p* < 0.05. Exploratory comparisons of BHI across treatment categories were performed using Welch’s ANOVA, followed by Bonferroni post hoc pairwise comparisons corrected for multiple testing. ANCOVA was used to examine whether treatment-category differences in BHI persisted after adjustment for EDSS. There were no missing data for BHI or cognitive outcomes; therefore, complete-case analyses were performed. No formal sample-size calculation was performed; sample size was determined by feasibility and consecutive recruitment during the study period.

## Results

Ninety RRMS patients and 45 age- and sex-matched HC were included. A flow diagram detailing participant screening and exclusions is provided in Online Resource 1 (Supplementary Fig. 1). The two groups were well matched for age, sex and smoking status (Table 1). All patients, except one, were receiving DMT at the time of evaluation. The primary analysis compared BHI between RRMS patients and HC and secondary analyses evaluated relationships with disability and cognition.

**Table 1:** Baseline demographics and clinical characteristics of the study population

	MS (*n* = 90)	Controls (*n* = 45)	*p* value
Age (years)	39.7 ± 9.03	39.7 ± 9.32	1.000
Female sex, *n *(%)	61 (68%)	31 (69%)	0.896
Current smokers, *n* (%)	9 (10%)	5 (11.1%)	0.842
Disease duration (years)	7.0 (4.0-15.25)	—	—
EDSS score	1.75 (1.0–3.0)	—	—
Number of DMTs	2.0 (1.75-3.0)	—	—
Relapses last 2 years, *n* (%)	33 (36.7%)	—	—
Glatiramer Acetate	3		
Interferon beta	4		
Dimethyl Fumarate	8		
Teriflunomide	4		
Ozanimod	4		
Fingolimod	4		
Natalizumab	25		
Ofatumumab	7		
Ocrelizumab	25		
Cladribine	4		
Alemtuzumab	1		

Abbreviations: *MS* multiple sclerosis, *EDSS* expanded disability status scale, *DMTs *disease-modifying therapies

Age is shown as mean ± SD, whereas disease duration, EDSS, number of DMTs are shown as median (interquartile range)

Group comparisons were performed using independent-samples t-test or χ²/Fisher’s exact test, as appropriate

Counts do not sum to the total MS sample because one participant was untreated at the time of assessment

**Fig. 1:**
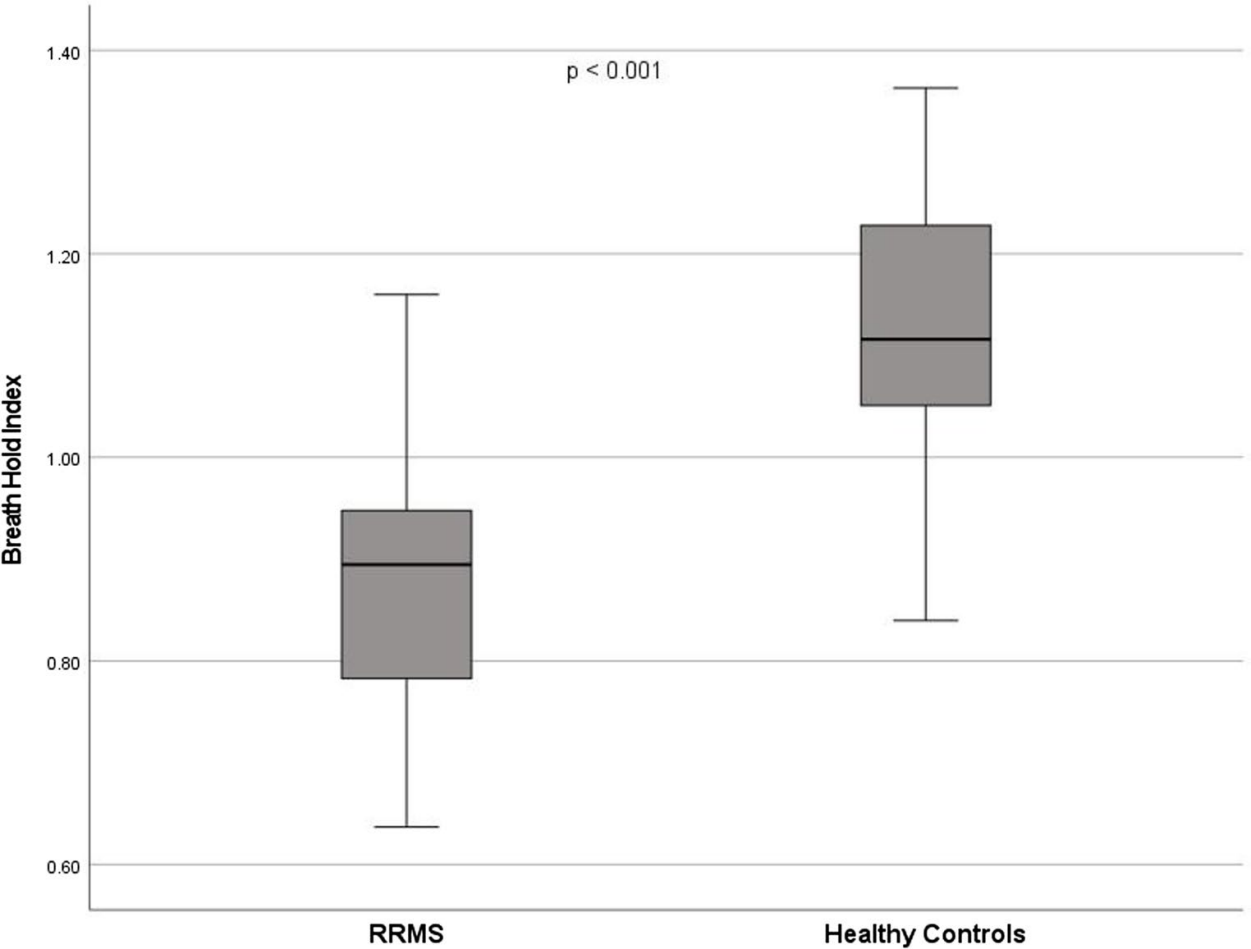
Cerebrovascular reactivity assessed by the Breath-Holding Index in patients with relapsing–remitting multiple sclerosis (RRMS) and healthy controls

BMFV and BH-MFV were lower in patients with RRMS than in HC (Table 2). CVR, assessed by the BHI, was lower in RRMS patients than in controls (0.88 ± 0.13 vs. 1.13 ± 0.13; mean difference − 0.256; 95% CI − 0.302 to − 0.209; *p* < 0.001) (Table 2; Fig. 1).

**Table 2:** Cerebrovascular reactivity measures in RRMS patients and healthy controls

	MS (*n* = 90)	Controls (*n* = 45)	*p* value	Cohen’s D
Baseline MFV (cm/s)	57.8 ± 10.2	63.2 ± 8.2	0.001*	-0.56
Breath-holding MFV (cm/s)	72.4 ± 13.7	84.3 ± 12.3	< 0.001	-0.66
Breath-Holding Index	**0.88 ± 0.13**	**1.13 ± 0.13**	**< 0.001**	**-1.99**

Abbreviations: *RRMS* relapsing–remitting multiple sclerosis, *MFV *mean flow velocity

Values are presented as mean ± standard deviation

*Welch’s *t*-test was applied due to unequal variances (Levene’s test *p* < 0.05)

Effect sizes are reported as Cohen’s *d* (negative values indicate lower values in the MS group)

**Fig. 2:**
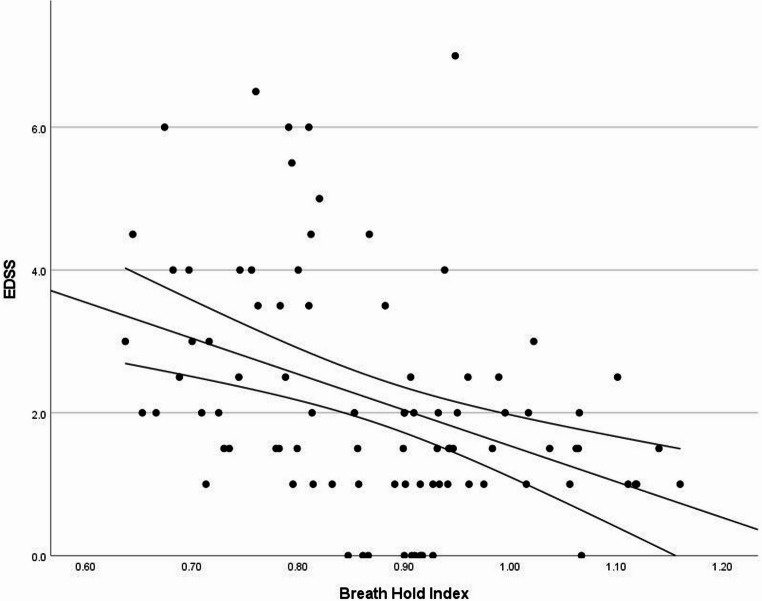
Scatterplot illustrating the association between cerebrovascular reactivity, assessed by the Breath-Holding Index (BHI), and neurological disability (Expanded Disability Status Scale, EDSS) in patients with relapsing–remitting multiple sclerosis. The solid line represents the fitted linear regression line and is shown for visualization purposes only. Spearman’s ρ = −0.420 (95% CI − 0.581 to − 0.228; *p* < 0.001)

To further characterize the control group, we examined the association between BHI and age among healthy controls. In healthy controls, BHI was inversely associated with age (*r* = − 0.459; 95% CI − 0.663 to − 0.192; *p* = 0.002). Disability and cognitive assessments were not available for HC; therefore, analyses involving EDSS and BICAMS performance were restricted to the RRMS cohort. Within the MS cohort, lower BHI values were associated with increasing age (*r* = − 0.353; 95% CI − 0.527 to − 0.152; *p* = 0.001) and with greater neurological disability (EDSS; ρ=−0.420; 95% CI − 0.581 to − 0.228; *p* < 0.001) (Fig. 2). In contrast, no associations were observed with disease duration, number of DMTs, or recent relapses.

Hierarchical linear regression analyses were conducted to examine the relationship between BHI values and EDSS scores after adjustment for demographic and disease-related factors (Online Resource 1, Supplementary Table 1). Age and disease duration were entered a priori and explained 25.3% of the variance in EDSS (R² = 0.253; *p* < 0.001). In Model 2, the addition of the BHI improved model fit (ΔR² = 0.051; *p* = 0.014), and lower BHI values were independently related to higher EDSS scores (B = − 3.07; 95% CI − 5.52 to − 0.63). In Model 3, which additionally included number of DMTs and relapses in the previous 2 years, this relationship persisted (B = − 3.51; 95% CI − 5.82 to − 1.20; *p* = 0.003), whereas disease duration and recent relapses were not independently related to EDSS. The final model explained 40.1% of EDSS variance (adjusted R² = 0.365). Sensitivity analyses excluding number of DMTs yielded consistent results, with BHI remaining related to EDSS (B = − 3.19; 95% CI − 5.61 to − 0.78; *p* = 0.010). Residual diagnostics indicated no violations of linear regression assumptions.

**Fig. 3:**
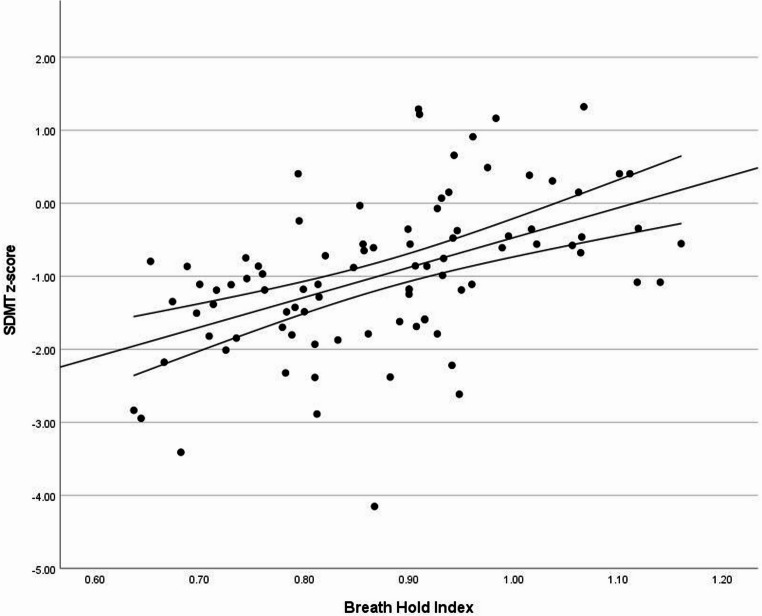
Scatterplot illustrating the association between cerebrovascular reactivity, assessed by the Breath-Holding Index (BHI), and processing speed (Symbol Digit Modalities Test [SDMT] z-score) in patients with relapsing–remitting multiple sclerosis. The solid line represents the fitted linear regression line and is shown for visualization purposes only. Pearson’s *r* = 0.503 (95% CI 0.331 to 0.643; *p* < 0.001)

Within the MS cohort, higher BHI values were observed with better processing speed as measured by the SDMT z-score (*r* = 0.503; 95% CI 0.331–0.643) (Fig. 3) and with BVMT-R z-score (*r* = 0.356; 95% CI 0.161–0.525). In contrast, no statistically meaningful relationship was identified between BHI and GVLT z-score (*r* = 0.087; 95% CI − 0.122 to 0.289). Cognitive z-scores were approximately normally distributed; therefore, Pearson correlation analyses were applied (Table 3). Associations with SDMT and BVMT-R remained significant after Bonferroni correction for three cognitive comparisons (adjusted threshold *p* < 0.017).

**Table 3:** Associations between cerebrovascular reactivity and cognitive performance in patients with multiple sclerosis

Cognitive test	Pearson’s *r*	95% CI	*p* value
SDMT z-score	0.503	0.331–0.643	< 0.001
GVLT z-score	0.087	-0.122–0.289	0.414
BVMT-R z-score	0.356	0.161–0.525	< 0.001

Abbreviations: *SDMT* symbol digit modalities test, *GVLT *greek verbal learning test, *BVMT-R* brief visuospatial memory test–revised

Values are Pearson correlation coefficients with 95% confidence intervals

Hierarchical linear regression analyses were subsequently restricted to the SDMT. After adjustment for EDSS scores and disease duration, BHI remained independently related to SDMT performance (B = 3.69; 95% CI 2.11 to 5.26; *p* < 0.001), explaining an additional proportion of variance beyond clinical covariates (ΔR² = 0.17) (Online Resource 1, Supplementary Table 2). Disease duration was independently associated with SDMT performance in adjusted models. In sensitivity analysis additionally adjusting for recent relapse activity, the relationship between BHI and SDMT remained unchanged. In secondary adjusted analyses, BHI was also independently related to visuospatial memory (BVMT-R z-score; ΔR² = 0.168; *p* < 0.001), whereas adding recent relapses did not improve model fit (Online Resource 1, Supplementary Table 3).

In an exploratory analysis according to current DMT category, patients were grouped into first-line therapies, natalizumab, and anti-CD20 therapies (Online Resource 1, Supplementary Table 4). First-line therapies included glatiramer acetate, interferons, teriflunomide, dimethyl fumarate, and ozanimod, whereas anti-CD20 therapies included ocrelizumab and ofatumumab. Patients receiving other treatments or no treatment were excluded from this analysis because of small subgroup sizes and heterogeneity of treatment mechanisms. BHI was numerically highest in the natalizumab group (first-line: 0.860 ± 0.135; natalizumab: 0.936 ± 0.124; anti-CD20: 0.862 ± 0.118), but the overall between-group difference did not reach statistical significance (Welch’s F [2, 47.89] = 3.045, *p* = 0.057). No pairwise comparison remained significant after correction for multiple testing. After adjustment for EDSS, no significant differences in BHI were observed across treatment categories (ANCOVA, *p* = 0.166).

## Discussion

The main findings of our study are twofold. First, patients with RRMS exhibited significantly reduced CVR, as assessed by the BHI, compared with HC, indicating impaired cerebral hemodynamic responses in MS. Second, within the MS cohort, lower CVR was independently associated with greater neurological disability and poorer cognitive performance, particularly in the domain of information processing speed. These findings support a link between vascular dysfunction, disability burden, and cognition, and reinforce the concept that impaired vascular reserve represents a clinically relevant component of MS pathophysiology beyond focal inflammatory activity.

CVR reflects the vasodilatory reserve of cerebral arterioles in response to vasoactive stimuli and supports stable cerebral perfusion both at rest and during neuronal activation (neurovascular coupling), thereby compensating for metabolic fluctuations and systemic hemodynamic changes [8].

MRI perfusion studies consistently demonstrate diffuse cerebral hypoperfusion in MS, involving normal-appearing white matter and cortical gray matter, and detectable even in early RRMS despite preserved volumes, with lower perfusion associated with worse memory performance [17]. Reduced perfusion has been associated with greater disability and worse cognitive performance and appears largely independent of overt tissue atrophy, supporting a diffuse microvascular process rather than a secondary consequence of structural loss [18]. Spatial analyses further link hypoperfusion to lesion vulnerability, with chronic plaques preferentially accumulating in low-perfusion white-matter territories—particularly in secondary progressive disease—and persistent T1-hypointense lesions clustering almost exclusively in such regions [19, 20]. Consistent with these in-vivo findings, neuropathological mapping revealed preferential accumulation of demyelination and retrograde neurodegeneration within arterial watershed zones of intrinsically low blood supply, reinforcing the concept that reduced perfusion promotes irreversible tissue injury [21]. Taken together, these findings provide a biological framework for the reduced CVR observed in RRMS. Impaired vasodilatory reserve may hinder flow stabilization and promote chronic hypoperfusion, particularly in vulnerable watershed regions, thereby amplifying inflammatory and metabolic stress, impairing repair, and favoring persistent tissue injury. Within this context, the association of lower CVR with greater disability and slower information processing suggests that vascular dysfunction may contribute to lesion accumulation and network disruption in MS.

In our study, reduced CVR was independently associated with greater neurological disability, with BHI explaining additional EDSS variance beyond age and disease duration and remaining significant after adjustment for recent relapses and treatment exposure, indicating that vascular dysfunction relates more closely to accumulated disability than to overt inflammatory activity. This pattern closely mirrors contemporary large-scale clinical datasets demonstrating that disability progression in RRMS is frequently driven by progression independent of relapse activity (PIRA), rather than relapse-associated worsening [22, 23]. Furthermore, mechanistic syntheses highlight chronic compartmentalized inflammation, mitochondrial dysfunction, energy failure, and impaired remyelination as central drivers of PIRA [24]. Within this framework, although causal inference cannot be established, our finding that CVR impairment correlates with EDSS, but not recent relapses or treatment proxies supports the hypothesis that reduced vascular reserve may contribute to the biological substrate of PIRA by amplifying diffuse neurodegenerative processes that proceed independently of focal inflammatory activity.

Cognitive impairment is common across all disease stages of MS and predominantly affects information processing speed and memory, domains routinely captured by BICAMS, with SDMT representing its most sensitive component [25]. Systematic reviews across neurological disorders further identify impaired CVR as a determinant of cognitive performance [26]. Previous hypercapnia MRI studies have demonstrated reduced CVR in MS compared with controls within cognition-relevant functional networks, suggesting network-level blood-flow regulation abnormalities [8] Interestingly, another work has shown that lower extracranial cerebral arterial blood flow was associated with poorer SDMT and BVMT-R performance and proposed that early network inefficiency in MS is initially counterbalanced by increased CBF recruitment to preserve cognitive performance; however, in the setting of reduced total extracranial arterial inflow, maintenance of adequate intracerebral perfusion would critically depend on intact CVR [27]. Our findings extend this framework by demonstrating that CVR is indeed impaired in RRMS and independently, strongly associated with cognitive performance, particularly processing speed as measured by the SDMT - the most sensitive cognitive test in MS - while remaining independent of disability, disease duration, and recent relapse activity. These results support failure of compensatory hemodynamic mechanisms as a contributor to cognitive dysfunction in MS. Mechanistically, impaired CVR may reflect dysfunction of the neurovascular unit (NVU) and reduced vasodilatory reserve, limiting the capacity to match regional blood flow to metabolic demand and thereby amplifying “energy failure” during cognitively demanding tasks.

The mechanisms underlying impaired CVR in MS remain incompletely understood and are likely multifactorial. Chronic neuroinflammation may disrupt endothelial signaling and vasomotor control through cytokine-mediated injury and oxidative stress, thereby limiting nitric oxide–dependent dilation and increasing vascular stiffness [11, 28]. In this context, sustained NO overproduction driven by oxidative stress may induce vascular habituation through endothelial and smooth muscle desensitization, downregulation of NO signaling, and ultimately reduced CVR [29]. Conversely, reactive oxygen species may instead scavenge NO and blunt endothelial-mediated dilation [10]. Astrocytic dysfunction represents another plausible contributor: reactive astrocytes within MS lesions release endothelin-1, a potent vasoconstrictor associated with cerebral hypoperfusion and reversible flow reductions following endothelin antagonism [30], while broader astroglial pathology may further impair flow regulation [2].

An additional, non-mutually exclusive interpretation is that reduced CVR reflects impaired cerebral autoregulatory control. Cerebral autoregulation depends on the integrated function of vascular smooth muscle, endothelial signaling, neurovascular coupling, and autonomic modulation of cerebrovascular tone. In MS, inflammatory demyelinating lesions may disrupt components of the central autonomic network, including brainstem autonomic nuclei and their supratentorial connections with the insula, anterior cingulate cortex, hypothalamus, amygdala, and prefrontal regions. Such disruption may alter sympathetic–parasympathetic balance and impair the dynamic regulation of cerebral blood flow during hypercapnic or metabolic stress [31]. In line with this concept, recent TCD evidence suggests that more efficient dynamic cerebral autoregulation is associated with more preserved brain structure and better cognitive performance in MS, supporting the clinical relevance of autoregulatory mechanisms in this disease [32].

Accordingly, impaired CVR in RRMS may arise from both peripheral/neurovascular mechanisms, including endothelial and astrocytic dysfunction, and central mechanisms involving altered autonomic network connectivity and cerebral autoregulatory failure. These mechanisms are not mutually exclusive and may interact to reduce cerebrovascular adaptability, impair neurovascular coupling, and promote chronic hypoperfusion or energetic insufficiency during increased metabolic demand. Within this framework, the observed association of lower CVR with greater disability and poorer cognitive performance supports the view that impaired vascular reserve may constitute a mechanistically relevant link between vascular dysregulation and neurodegenerative processes in MS.

TCD studies assessing CVR in MS have reported heterogeneous findings, likely reflecting differences in methods, physiological stimuli, disease phenotypes, and comparator groups. Several negative studies employed a shorter 15-s breath-holding protocol, which may reduce the magnitude of CO₂ stimulus compared with the conventional 30-s breath holding, potentially attenuating group differences [33]. Differences in comparator populations further complicate interpretation, as some studies compared patients with multiple sclerosis to migraine controls who also exhibited white-matter lesions, a design that may obscure disease-specific effects on vasomotor reserve [34]. Conversely, other work has shown reduced BHI in MS compared with controls but without significant correlations with age, EDSS, or disease duration [35]. Using a similar TCD breath-holding protocol subsequent investigations reported markedly reduced CVR in RRMS compared with healthy controls (BHI 0.87 vs. 1.15), values concordant with those observed in our study and suggested secondary progressive MS phenotype as an independent predictor of reduced CVR [36]. These findings provide further external validation of our observations and support impaired CVR as a core vascular feature of RRMS. Additional studies have observed lower BHI in MS than controls that did not reach statistical significance, but crucially demonstrated significant endothelial dysfunction in patients with MS as assessed by flow-mediated dilation, highlighting systemic endothelial dysfunction as a feature of MS and providing a biological framework upon which our direct in-vivo observations are based [37]. Finally, recent work highlighted that cerebral hemodynamic regulation abnormalities in MS may also manifest as impaired cerebral autoregulation (CA), with autoregulatory indices relating to cognitive outcomes, underscoring that distinct TCD-derived vascular constructs (CVR versus CA) may capture complementary aspects of neurovascular dysfunction [32].

MRI studies using hypercapnic challenges have provided convergent but heterogeneous evidence for altered cerebrovascular regulation in MS. Using ASL-based CO₂ inhalation, prior work has demonstrated diffuse reductions in gray-matter and network-level CVR in MS compared with controls, with CVR correlating with lesion burden and gray-matter atrophy, hypothesizing a link between vascular dysfunction and neurodegeneration [8]. Other investigations, in contrast, did not find group-level CVR differences but reported significantly lower CVR in cognitively impaired compared with cognitively preserved MS patients, suggesting that CVR may relate more closely to cognitive status than to diagnosis alone [38]. Importantly, studies employing combined BOLD–CBF hypercapnic MRI have shown that impaired arterial compliance along the cerebrovascular tree leads to neurovascular uncoupling and reduced CVR during neural activation, with arterial CVR independently predicting processing speed (as measured by reaction time) after adjustment for lesion burden, gray-matter volume, and disability [39]. Our TCD findings extend these frameworks at the bedside by showing that reduced BHI is clinically relevant, being associated with SDMT/BVMT performance and disability within RRMS. Furthermore, expanding the pathophysiological mechanisms, a recent study showed that CVR alterations in MS are predominantly localized along white-matter pathways and described a “blood-steal” phenomenon whereby relatively preserved vessels divert flow away from vasodilatory-impaired regions, resulting in functional hypoperfusion of affected tracts and providing a mechanistic link between microvascular dysfunction, structural disconnection, and cognitive impairment [40].

In summary, this study demonstrates that patients with RRMS exhibit significantly impaired CVR compared with healthy individuals and that impaired CVR is independently associated with neurological disability and cognitive performance, particularly processing speed. By integrating TCD-derived BHI with clinical and cognitive measures, this study extends the existing literature and identifies impaired vascular reserve as a potentially relevant pathophysiological component of MS, plausibly mediated by interacting mechanisms involving endothelial and NVU dysfunction, autonomic network disruption and impaired cerebral autoregulatory control, with clinical correlates in EDSS and cognition. We hypothesize that CVR impairment may lead to inefficient blood-flow recruitment during metabolic demand, thereby promoting neuronal dysfunction and loss, and contribute to PIRA, cognitive decline, and the diffuse neurodegenerative processes characteristic of MS. These observations may support CVR as a candidate biomarker of diffuse disease burden and relapse-independent progression.

Key strengths of this study include a large TCD-based assessment of CVR in clinically stable RRMS patients, careful matching with controls, exclusion of vascular comorbidities, and assessment during clinical stability to minimize relapse/steroid confounding. The relatively young and vascular-risk–free cohort enhances the specificity of the findings by minimizing confounding from age-related or systemic vascular pathology, allowing clearer interpretation of MS-related influences on CVR. The standardized, clinically feasible breath-holding protocol with repeated bilateral measurements enhances reproducibility and translational potential. The low-cost, non-invasive nature of TCD supports scalability for multicenter and longitudinal use. Limitations include the cross-sectional design, which precludes causal inference, and the operator dependence and velocity-based nature of TCD. We did not include direct autonomic function measures, such as heart-rate variability spectral analysis or deep-breathing respiratory sinus arrhythmia; therefore we could not directly assess the contribution of autonomic dysregulation to impaired CVR. The absence of concurrent MRI data is another limitation, as lesion burden, atrophy, perfusion abnormalities, and lesion topography could not be evaluated. In particular, brainstem or other central autonomic network lesions may differentially affect cerebrovascular regulation. Finally, because the study was restricted to RRMS, the findings cannot be generalized to progressive MS phenotypes. Future longitudinal studies integrating TCD- and MRI-based CVR with structural, perfusion and functional imaging, lesion evolution, and cognitive trajectories will be required to determine whether impaired vascular reserve precedes progression and predicts PIRA or cognitive decline. Interventional trials targeting endothelial function or cerebrovascular health may further clarify causality and open therapeutic avenues. Clinically, incorporation of CVR metrics into risk-stratification frameworks could help identify patients vulnerable to insidious progression and cognitive decline beyond overt inflammatory activity.

## Supplementary Information

Below is the link to the electronic supplementary material.

Supplementary Material 1

Supplementary Material 2

## Data Availability

The datasets generated and analyzed during the current study are not publicly available due to ethical and data protection restrictions but are available from the corresponding author upon reasonable request.
