# Supplementary material for: Impaired cerebrovascular reactivity is associated with disability and cognitive performance in relapsing–remitting multiple sclerosis
Source: Neurol Sci. 2026 Jun 9;47(7):558. doi: 10.1007/s10072-026-09163-5 (PMC13249697; doi:10.1007/s10072-026-09163-5)
Supplement: Supplementary file 2 — Supplementary Material 2 [file 10072_2026_9163_MOESM2_ESM.docx]

**Supplementary Material for:**

Impaired Cerebrovascular Reactivity Is Associated With Disability and Cognitive Performance in Relapsing–Remitting Multiple Sclerosis

**Journal: Neurological Sciences**

Evangelos Ntais^1^, Vasiliki Kostadima^2^, Andriana Haski^2^, Konstantinos Tsamis^3^

Sotirios Giannopoulos^4^, Spyridon Konitsiotis^1^

**Affiliations**

^1^ Department of Neurology, Faculty of Medicine, University of Ioannina, Ioannina, Greece

^2^ Department of Neurology, University General Hospital of Ioannina, Ioannina, Greece

^3^ Department of Physiology, Faculty of Medicine, University of Ioannina, Ioannina, Greece

^4^ Second Department of Neurology, "Attikon" University Hospital, School of Medicine, National and Kapodistrian University of Athens, Athens, Greece

**Supplementary Figure 1**

**Participant Flow Diagram**


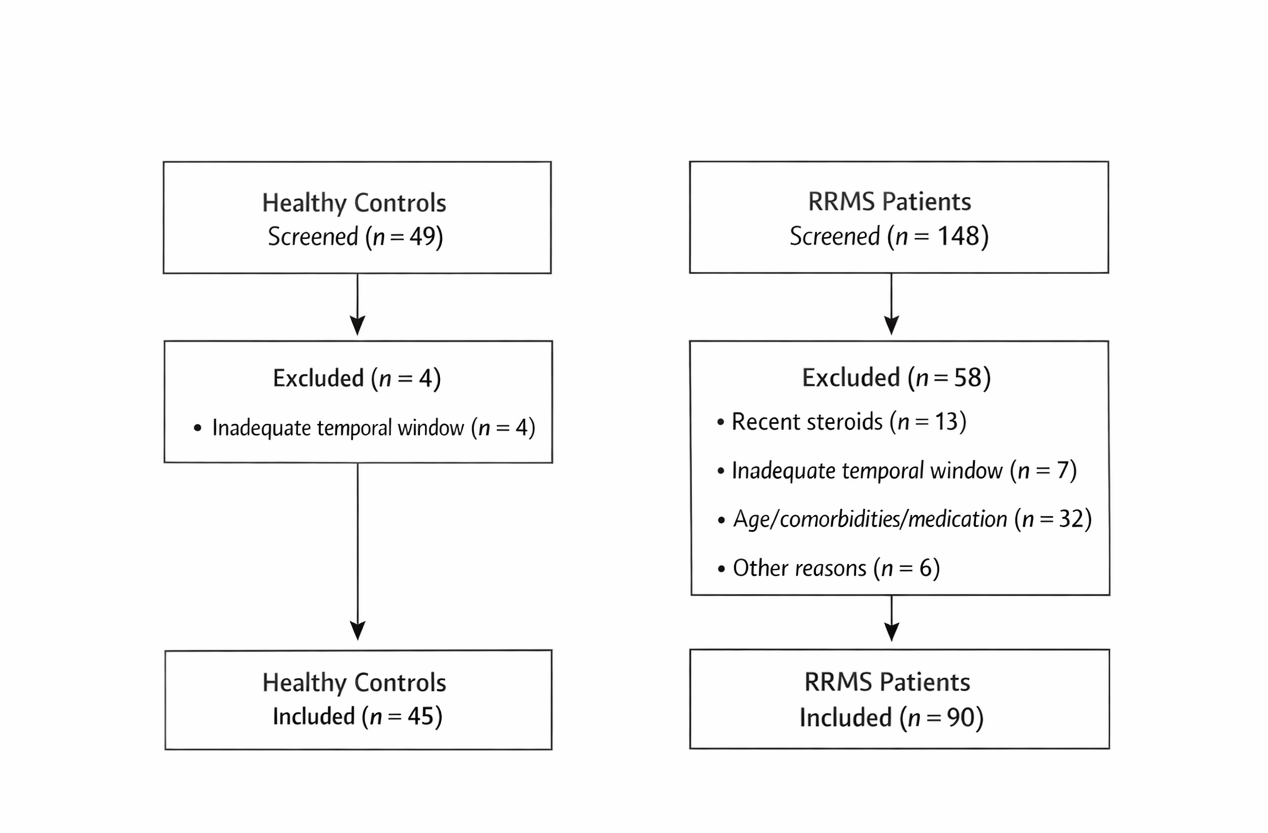


| **Supplementary Table 1. Hierarchical linear regression models examining the association between cerebrovascular reactivity and neurological disability (EDSS) in RRMS** | | | | | | |
| --- | --- | --- | --- | --- | --- | --- |
| **Predictor** | **Model 1 B**  **(95% CI)** | **p value** | **Model 2 B**  **(95% CI)** | **p value** | **Model 3 B**  **(95% CI)** | **p value** |
| Age | 0.08  (0.04 to 0.11) | <0.001 | 0.06  (0.03 to 0.1) | 0.001 | 0.07  (0.03 to 0.1) | <0.001 |
| Disease Duration | 0.03  (-0.02 to 0.07) | 0.221 | 0.03  (-0.02 to 0.07) | 0.263 | -0.04  (-0.09 to 0.02) | 0.183 |
| BHI |  |  | -3.07  (-5.52 to -0.63) | 0.014 | -3.51  (-5.83 to -1.2) | 0.003 |
| Number of DMTs |  |  |  |  | 0.36 (0.13 to 0.59) | 0.002 |
| Relapses (last 2 years) |  |  |  |  | -0.53  (-1.14 to 0.08) | 0.089 |
| R^2^ | 0.253 |  | 0.304 |  | 0.401 |  |
| ΔR^2^ | - |  | 0.051 |  | 0.097 |  |
| Adjusted R^2^ | 0.236 |  | 0.280 |  | 0.365 |  |

Abbreviations: BHI = Breath-Holding Index; DMT = disease-modifying therapy; EDSS = Expanded Disability Status Scale.

Values are unstandardized regression coefficients (B) with 95% confidence intervals.
EDSS was modeled as a continuous outcome.

Model 1 included age and disease duration.
Model 2 additionally included Breath-Holding Index.
Model 3 additionally included number of disease-modifying therapies and relapses in the previous 2 years.

Residual diagnostics indicated no violation of linear regression assumptions.

| **Supplementary Table 2. Hierarchical linear regression models examining the association between cerebrovascular reactivity and SDMT z-score in RRMS** | | | | | | |
| --- | --- | --- | --- | --- | --- | --- |
| **Predictor** | **Model 1 B**  **(95% CI)** | **p value** | **Model 2 B**  **(95% CI)** | **p value** | **Model 3 B**  **(95% CI)** | **p value** |
| EDSS | -0.26  (-0.39 to -0.13) | <0.001 | -0.15  (-0.28 to -0.02) | 0.020 | -0.15  (-0.28 to -0.02) | 0.027 |
| Disease Duration | 0.02  (-0.01 to 0.05) | 0.119 | 0.03  (0.01 to 0.06) | 0.031 | 0.03  (0.01 to 0.06) | 0.030 |
| BHI |  |  | 3.69  (2.11 to 5.26) | <0.001 | 3.73  (2.14 to 5.33) | <0.001 |
| Relapses (last 2 years) |  |  |  |  | 0.09  (-0.32 to 0.51) | 0.655 |
| R^2^ | 0.147 |  | 0.319 |  | 0.320 |  |
| ΔR^2^ | - |  | 0.172 |  | 0.002 |  |
| Adjusted R^2^ | 0.128 |  | 0.295 |  | 0.288 |  |

Abbreviations: SDMT= Symbol Digit Modalities Test; BHI = Breath-Holding Index; EDSS = Expanded Disability Status Scale.

Values are unstandardized regression coefficients (B) with 95% confidence intervals.

Model 1 included EDSS and disease duration.
Model 2 additionally included Breath-Holding Index.
Model 3 additionally included relapses in the previous 2 years.

Residual diagnostics indicated no violation of linear regression assumptions.

| **Supplementary Table 3. Hierarchical linear regression models examining the association between cerebrovascular reactivity and BVMT-R z-score in RRMS** | | | | | | |
| --- | --- | --- | --- | --- | --- | --- |
| **Predictor** | **Model 1 B**  **(95% CI)** | **p value** | **Model 2 B**  **(95% CI)** | **p value** | **Model 3 B**  **(95% CI)** | **p value** |
| EDSS | -0.01  (-0.21 to 0.19) | 0.905 | 0.14  (-0.06 to 0.33) | 0.176 | 0.13  (-0.07 to 0.33) | 0.197 |
| Disease Duration | 0.02  (-0.02 to 0.06) | 0.337 | 0.03  (-0.01 to 0.07) | 0.149 | 0.03  (-0.02 to 0.07) | 0.204 |
| BHI |  |  | 5.08  (2.67 to 7.49) | <0.001 | 5.04  (2.60 to 7.48) | <0.001 |
| Relapses (last 2 years) |  |  |  |  | -0.08  (-0.72 to 0.56) | 0.248 |
| R^2^ | 0.011 |  | 0.179 |  | 0.179 |  |
| ΔR^2^ | - |  | 0.168 |  | 0.001 |  |
| Adjusted R^2^ | -0.012 |  | 0.150 |  | 0.141 |  |

Abbreviations: BVMT-R = Brief Visuospatial Memory Test–Revised; BHI = Breath-Holding Index; EDSS = Expanded Disability Status Scale.

Values are unstandardized regression coefficients (B) with 95% confidence intervals.

Model 1 included EDSS and disease duration.
Model 2 additionally included Breath-Holding Index.
Model 3 additionally included relapses in the previous 2 years.

Residual diagnostics indicated no violation of linear regression assumptions.

| **Supplementary Table 4. Baseline characteristics according to treatment category** | | | |
| --- | --- | --- | --- |
|  | **First Line = 23** | **Natalizumab = 25** | **Anti-CD20 = 32** |
| **Age** | 37.5 ±10 | 37.5 ±9.4 | 41.7 ±7.9 |
| **Sex (female)** | 60.9% | 88% | 62.5% |
| **Disease Duration** | 4 (2-8) | 11 (5-19) | 7 (3.5-13.75) |
| **EDSS** | 1.5 (1-2) | 1.5 (1-2) | 3.25 (1.5-4) |

*Values are presented as mean ± SD, median (interquartile range), or n (%), as appropriate. First-line therapies included glatiramer acetate, interferons, teriflunomide, dimethyl fumarate, and ozanimod. Anti-CD20 therapies included ocrelizumab and ofatumumab. Patients receiving other treatments or no treatment were excluded from this exploratory analysis because of small subgroup sizes and heterogeneity of treatment mechanisms. The table is provided for descriptive purposes; no formal between-group comparisons of baseline variables were prespecified.*
